# Repositioning Mifepristone as a Leukaemia Inhibitory Factor Receptor Antagonist for the Treatment of Pancreatic Adenocarcinoma

**DOI:** 10.3390/cells11213482

**Published:** 2022-11-03

**Authors:** Cristina Di Giorgio, Antonio Lupia, Silvia Marchianò, Martina Bordoni, Rachele Bellini, Carmen Massa, Ginevra Urbani, Rosalinda Roselli, Federica Moraca, Valentina Sepe, Bruno Catalanotti, Elva Morretta, Maria Chiara Monti, Michele Biagioli, Eleonora Distrutti, Angela Zampella, Stefano Fiorucci

**Affiliations:** 1 Department of Medicine and Surgery, University of Perugia, 06123 Perugia, Italy; 2 Department of Pharmacy, University of Naples Federico II, 80131 Naples, Italy; 3 Campus Salvatore Venuta, Net4Science Srl, University “Magna Græcia”, Viale Europa, 88100 Catanzaro, Italy; 4 Department of Pharmacy, University of Salerno, 84084 Salerno, Italy; 5 Azienda Ospedaliera di Perugia, 06121 Perugia, Italy

**Keywords:** LIF/LIFR axis, pancreatic cancer, mifepristone, cyclins, cell cycle, gemcitabine, repositioning

## Abstract

Pancreatic cancer is a leading cause of cancer mortality and is projected to become the second-most common cause of cancer mortality in the next decade. While gene-wide association studies and next generation sequencing analyses have identified molecular patterns and transcriptome profiles with prognostic relevance, therapeutic opportunities remain limited. Among the genes that are upregulated in pancreatic ductal adenocarcinomas (PDAC), the leukaemia inhibitory factor (LIF), a cytokine belonging to IL-6 family, has emerged as potential therapeutic candidate. LIF is aberrantly secreted by tumour cells and promotes tumour progression in pancreatic and other solid tumours through aberrant activation of the LIF receptor (LIFR) and downstream signalling that involves the JAK1/STAT3 pathway. Since there are no LIFR antagonists available for clinical use, we developed an in silico strategy to identify potential LIFR antagonists and drug repositioning with regard to LIFR antagonists. The results of these studies allowed the identification of mifepristone, a progesterone/glucocorticoid antagonist, clinically used in medical abortion, as a potent LIFR antagonist. Computational studies revealed that mifepristone binding partially overlapped the LIFR binding site. LIF and LIFR are expressed by human PDAC tissues and PDAC cell lines, including MIA-PaCa-2 and PANC-1 cells. Exposure of these cell lines to mifepristone reverses cell proliferation, migration and epithelial mesenchymal transition induced by LIF in a concentration-dependent manner. Mifepristone inhibits LIFR signalling and reverses STAT3 phosphorylation induced by LIF. Together, these data support the repositioning of mifepristone as a potential therapeutic agent in the treatment of PDAC.

## 1. Introduction

Pancreatic cancer (PC) is the seventh leading cause of cancer-related deaths in industrialized countries and is projected to become the second leading cause of cancer death worldwide by 2030 [1]. PC includes several histological subtypes; however, pancreatic ductal adenocarcinoma (PDAC) is responsible for ≈85% of total PC [2]. The majority of cases of PDAC are thought to arise from microscopic precursor lesions called pancreatic intraepithelial neoplasia (PanIN), which in the vast majority of cases are below the detectable size threshold of current clinical imaging modalities. In addition, a minority of cases of PDCA might arise from intraductal papillary mucinous neoplasms (IPMNs) and mucinous cystic neoplasms. The main reason for the dismal prognosis of PDAC is a late diagnosis, since ≈90% of cancer is detected after spreading beyond the pancreas, with systemic metastases occurring in >50% of patients at the time of diagnosis [3,4] and only 20% of patients meeting the criteria for surgery at the time of diagnosis [1,5,6]. PDAC is a multifactorial disease driven by multiple environmental factors, including smoking and excessive alcohol consumption, obesity, diabetes mellitus and chronic pancreatitis, and, at least in part, by somatic mutations in oncogenes and tumour suppressor genes, such as *KRAS* along with the tumour suppressor genes *CDKN2A*, *TP53*, and *SMAD4* [7,8,9].

The clinical management of PDAC remains suboptimal, and although surgery in combination with adjuvant chemotherapy shows efficacy for stage I and II, only 10–15% of patients are diagnosed with resectable disease [10]. For the treatment of borderline and locally advanced tumours, the treatment with systemic neoadjuvant with or without radiation, followed by assessment for surgery, offers a 5-year survival rate of 10–15% [11,12]. The standard first-line treatment for stage I and II remains FOLFOX or FOLFIRINOX or gemcitabine plus albumin-bound (nab) paclitaxel [13]. Precision therapies designed to target specific mutations, including PD1 and PD-L1 in patients with microsatellite instability, are under evaluation [8]. Unfortunately, up to 50% of patients present a metastatic disease at the time of diagnosis, with a 5-year survival <5%. Additionally, PDAC patients might develop chemo-resistance, making the understanding of PDCA and the identification of novel molecular biomarkers and therapeutic targets urgent needs.

While the genetic subtyping of PDAC has shown poor clinical translational relevance, next generation sequencing studies have identified multiple gene transcripts that correlate with the patient’s survival. Leukaemia Inhibitory Factor (LIF) has been identified as one of the potential biomarkers of poor prognosis in patients with PDAC and is also a potential target for pharmacological intervention. LIF, a pleiotropic cytokine that belongs to the Interleukin (IL)-6 family [14], is overexpressed in a variety of solid tumours, including pancreas [15], stomach [16], liver [17], colon [18] and breast [19], and promotes cancer cell proliferation, remodelling, invasiveness and epithelial-to-mesenchymal transition (EMT) [20]. In target tissues, LIF signalling is mediated by its binding to an heterodimeric receptor complex formed by the LIF-receptor (LIFR) and the glycoprotein (gp) 130 subunit of the IL-6 receptor [21]. Upon binding, LIFR phosphorylates downstream proteins in the JAK-STAT3 signalling cascade; the inhibition of LIFR signalling has shown beneficial effects on cell growth and tumour progression in several cancers [22]. Recently, a new LIFR antagonist that inhibits oncogenic signalling has been reported [23]. However, there are no LIFR antagonists approved for clinical use, making the identification and repositioning of existing drugs as LIFR antagonists a potential approach for the treatment of LIFR-expressing PDAC. Building on this concept, we performed a similarity screening of the FDA approved drug database using as query the structure of the LIFR antagonist EC359 and identified mifepristone (also known as RU-486) as a potential LIFR antagonist. Mifepristone is a broad steroid antagonist, endowed with anti-progesterone and anti-androgen activity, clinically approved for the induction of medical abortion [24]. Furthermore, mifepristone exerts anti-proliferative effects in a variety of cancers, including gastric, breast, prostate, endometrial, and ovarian cancers [25].

In the present study, we developed an in silico strategy to support the repositioning of mifepristone as a LIFR antagonist and confirmed the computational data by in vitro studies on pancreatic cancer cell lines. Our results demonstrate that while LIF promotes cancer cell proliferation and migration in a JAK-STAT3–dependent manner, these features are reversed by mifepristone [26]. Our results form the basis for the exploitation of mifepristone in the treatment of PDAC.

## 2. Materials and Methods

### 2.1. GSE196009 Data Sets

The GSE196009 series (https://www.ncbi.nlm.nih.gov/geo/query/acc.cgi?acc=GSE196009) accessed on 1 August 2022 includes gene expression profiles (RNA-seq analysis, Illumina HiSeq 2000) of fresh or frozen PDAC tissues and adjacent normal pancreatic tissue from 12 Japanese patients.

### 2.2. Pancreatic Cancer Cell Lines

Human pancreatic cell lines MIA-PaCa-2 and PANC-1 were purchased from ATCC (Manassas, VA, USA). These cells were grown in DMEM (Sigma-Merk Life Science S.r.l. Milan, Italy) medium supplemented with 10% Foetal Bovine Serum (FBS), 1% L-Glutamine, and 1% Penicillin/Streptomycin, in a humidified 5% CO_2_ atmosphere at 37 °C. Cells were free from Mycoplasma contamination as confirmed by a Mycoplasma PCR Detection (Sigma) test and were regularly passaged to maintain exponential growth and used from early passages (<10 passages after thawing). To perform all experiments, cells were plated and serum was starved for 24 h and stimulated for 24 h.

### 2.3. Real-Time PCR

The RNA was extracted from patient biopsies using the Trizol reagent (Invitrogen) and from cell lines using and Direct-zol™ RNA MiniPrep w/Zymo-Spin™ IIC Columns (Zymo Research, Irvine, CA, USA), according to the manufacturer’s protocol. After purification from genomic DNA by DNase-I treatment (ThermoFisher Scientific, Waltham, MA, USA), 2 µg of RNA from each sample was reverse-transcribed using FastGene Scriptase Basic Kit (Nippon Genetics, Mariaweilerstraße, Düren, Germania) in a 20 μL reaction volume. Finally, 50 ng cDNA was amplified in a 20 μL solution containing 200 nM of each primer and 10 μL of SYBR Select Master Mix (ThermoFisher Scientific). All reactions were performed in triplicate, and the thermal cycling conditions were as follows: 3 min at 95 °C, followed by 40 cycles at 95 °C for 15 s, 56 °C for 20 s, and 72 °C for 30 s, using a Step One Plus machine (Applied Biosystem). The relative mRNA expression was calculated accordingly to the 2^−ΔCt^ method. Primers used in this study were designed using the PRIMER3 (http://frodo.wi.mit.edu/primer3/, accessed on 1 May 2022) software using the NCBI database. RT-PCR primers used in this study for human samples and human cell lines were as follows (forward (for) and reverse (rev)):

- Cmyc (for TCGGATTCTCTGCTCTCCTC; rev TTTTCCACAGAAACAACATCG),
- Snail1 (for ACCCACACTGGCGAGAAG; rev TGACATCTGAGTGGGTCTGG),
- Vimentin (for TCAGAGAGAGGAAGCCGAAA; rev ATTCCACTTTGCGTTCAAGG),
- Cxcr4 (for AACGTCAGTGAGGCAGATGA; rev TGGAGTGTGACAGCTTGGAG).

### 2.4. Immunofluorescence (IF)

Immunofluorescence staining was achieved on PANC-1 and MIA PaCa-2 alone or stimulated with LIF 10 ng/mL, EC359 25 nM, and mifepristone 10 μM. Cells were plated on slides using cytospin. The spots obtained were fixed in methanol for 20 min and then were washed 3 times with phosphate buffered saline (PBS 1X).

Subsequently, cells were permeabilized and then incubated with blocking buffer (PBS 1X with 10% horse serum and 1% BSA) for 1 h at room temperature. Primary antibodies anti-LIF-R (ab235908), anti-Vimentin (ab92547), anti-Ki67 (ab16667) (Abcam, Cambridge, UK) were incubated overnight at 4 °C. The next day, after 3 washes with PBS 1X containing 0.1% Tween 20 (PBST), cells spotted were incubated with secondary antibody Goat Anti-Rabbit IgG H&L (Aleza Fluor 488) (ab150077) for LIF-R and vimentin and with Goat anti-Rabbit IgG (H + L) Cross-Adsorbed Secondary Antibody (Alexa Fluor™ 568) (Invitrogen, Thermofisher scientific, Waltham, MA, USA) for Ki-67, for 1 h at room temperature in the dark. After 3 washes with PBST, the nucleus was counterstained with DAPI 1X for 1 min in the dark, and the reaction was stopped with a final wash in PBS 1X for 5 min. Then, slides were mounted with ProLong Glass Antifade Mountant (P36980) (Invitrogen, Thermofisher scientific, Waltham, MA, USA), sealed with nail polish, and observed with a fluorescence microscope Olympus BX60.

### 2.5. Image Analysis

Ki-67 positive cell numbers and whole cell numbers (as background) were counted in fields at a magnification of 100X. The Ki-67 score is defined as the ratio of the number of positively stained cells to the total number of cells assessed.

### 2.6. Cell Proliferation Assay

The cell viability assay was done using the CellTiter 96 Aqueous One Solution Cell Proliferation Assay (Promega, Milano, Italy), a colorimetric method for accessing the number of viable cells in proliferation, as described previously [16]. MIA-PaCa 2 and PANC-1 cells were seeded in DMEM complete medium at 36 × 10^3^ cells/100 μLl well into a 96-well tissue culture plate. After 24 h, cells were serum-starved for 24 h and then were primed with the LIFR major ligand, LIF (0.5, 5, 10, 50, and 100 ng/mL) or only with a vehicle. In another experimental setting, MIA-PaCa 2 cells were triggered with LIF (10 ng/mL) plus LIFR antagonist EC359 (25, 50, 100, and 1000 nM) (MedChemExpress, NJ 08852, USA) and in a different setting, mifepristone (0.1, 1, 10, 20, 50 μM); then, cell proliferation was assessed as mentioned above. Absorbance was measured using a 96-well reader spectrophotometer (490 nm). In these experiments, each experimental setting was replicated ten-fold. For analysis, the well background readings with the medium alone were subtracted from the sample read-outs.

### 2.7. Protein Preparation of hLIFR and hLIF-hLIFR Homology Model

The X-ray crystal structures of the *human* LIFR, *h*LIFR (Uniprot ID Code: P42702, PDB IDs 3E0G [26]) and of the *mouse* LIFR/*human* LIF complex, *m*LIFR*-h*LIF (Uniprot ID Code: P42703, PDB ID: 2Q7N [27]) were retrieved from the *RCSB Protein Data Bank* (www.rcsb.org). The *h*LIF-*h*LIFR complex used for molecular dynamic simulations (MDs) was manually built after the superposition of the *h*LIFR (PDB ID 3E0G) on the *m*LIFR (PDB ID 2Q7N). Both *m*/*h*LIFR structures showed high sequence identity with a typically extended *zig-zag* conformation [27] (Supplementary Figure S3, panel A). Hence, homology modelling was performed by replacing the *m*LIFR with the *h*LIFR (PDB ID: 3E0G [26]) in the *h*LIF-*m*LIFR structure (PDB ID: 2Q7N [27]). Sequence alignment analysis, performed on the domains D1–D5, suggests the following score values between *h*LIFR and *m*LIFR: 80%, 64%, and 77% for similarity, identity, and homology, respectively. Additionally, we observed that the principal contacts between *h*LIF (P51, K153, F156, and K159) and *m*LIFR (Q209, D210, G235, N256, G263, S262, and V278) [27] remain conserved even with respect to the complex *h*LIF-*h*LIFR (Supplementary Figure S3, panel A) (Table S1). All models were optimized by using the *Protein Preparation Wizard (PPw) tool* implemented in Maestro Suite 2021-1 (Schrödinger Release 2021-1) in order to assign bond orders, add hydrogen atoms, adjust disulphide bonds, add caps to chains break, and assign residues protonation state at pH 7.4.

### 2.8. FDA-Approved Library Preparation

The library of FDA-approved drugs was retrieved from DrugBank (www.drugbank.com) and prepared using the LigPrep (LigPrep. Schrödinger, release 2021–1, LigPrep; Schrödinger, LLC: New York, NY, USA, 2021) tool. Furthermore, the Epik (Schrödinger; Release 2021-1: Epik, S., LLC, New York, NY, USA, 2021) module was used to apply the protonation states at pH 7.4. The FDA library was then filtered by retaining only drugs with a molecular weight (MW) between 200 and 700 daltons, giving a total of 1852 molecules.

### 2.9. Docking Procedures

The X-ray structure of *h*LIFR (PDB IDs 3E0G [26]) was used for docking studies. Firstly, since LIF interacts with the central domains (D3-D4) of LIFR (Supplementary Figure S3, panel A,B), the SiteMap (SiteMap, S., LLC, New York, NY, USA, 2021) tool was used to search druggable cavities in that region. Secondly, based on the predicted site points, we set up the grid-box coordinates (Supplementary Figure S3, panel B), and the QM-Polarized Ligands Docking (QPLD) (Glide, S., LLC, New York, NY, USA, 2021; Jaguar, S., LLC, New York, NY, USA, 2021; QSite, S., LLC, New York, NY, USA, 2021) tool, available in Schrödinger Maestro Suite 2021-1, was used to execute docking calculations. The aim was to find out the correct electronic charges in protein-ligand docking, replace the charges by QM/molecular mechanical (MM) calculations, and treat only the ligands as the quantum region. This approach uses the QSITE (at the B3LYP/6-31G* level) and JAGUAR programs for the QM region and the IMPACT molecular modelling code for the MM region. Initially, a standard precision (SP) glide docking was carried out, generating ten poses, and submitted to QM-ESP charge calculation at the B3LYP/3-21G* level. The resulting poses were then re-docked for another run using the ESP atomic charges and SP scoring modes. Finally, the 20 best-docked poses per ligand were submitted to QM charge calculations using JAGUAR with accurate levels and ranked by G-Score value. Subsequently, after visual inspection, the best poses were refined with Induced Fit Docking (IFD) (Glide, S., LLC, New York, NY, USA, 2021; Prime, S., LLC, New York, NY, USA, 2021) in the “*extended sampling*” protocol. IFD offers better flexibility of the binding pocket residues and ligand throughout the docking. The ligand in the active site was used as the centroid to generate the grid files in default size (INNERBOX 10.0 Å). No constraints were applied, and a maximum of ten poses were saved after the docking process. To investigate the ligand and protein flexibility in the active site, the IFD extended sampling protocol was adopted. A maximum of 80 poses was generated, and the residues having at least one atom within 5 Å of ligand poses were subject to a conformational search and energy minimization process. The energy window for ligand conformational sampling was 2.5 kcal/mol. Again, to improve the analysis, the binding site was mapped with the SiteMap (SiteMap, S., LLC, New York, NY, USA, 2021) tool.

### 2.10. Molecular Dynamics Simulations (MDs)

Three different docking poses of mifepristone (complexes named: ID01, ID02-A, and ID02-B) were subjected to 100 ns of MDs in order to investigate their binding mode. Additionally, two other systems were simulated for comparison analysis: *(i) h*LIFR and *(ii) h*LIFR-LIF. MDs were conducted using the CUDA version implemented in the AMBER18 suite [28,29], with the Amber ff14SB force field [30] to treat the protein, while ligand charges were computed using the restrained electrostatic potential (RESP) fitting procedure [31]. Gaussian16 package [32] was used to calculate the ligand ESP using the 6-31G* basis set at the Hartree-Fock level of theory. Antechamber [33], coupled with the general amber force field (GAFF2) parameters [34], allowed RESP charges and the ligand force field parameters. After this initial step, each system was solvated in a 10 Å layer of the octahedral box using the transferable intermolecular potential with 3 point (TIP3P) [35] water molecules parameters and then neutralized by adding Na+ and Cl− ions. A cut-off of 9 Å was used for a non-bonded short-range interaction, while long-range electrostatic interactions were computed by means of the Particle Mesh Ewald (PME) [36] method using a 1.0 Å grid spacing in periodic boundary conditions. The SHAKE algorithm was used to constrain bonds involving hydrogen atoms with two fs integration time steps. Then, each system was minimized using the energy gradient convergence criteria set to 0.01 kcal/mol Å2 involving a multistep procedure: (1) only hydrogen atoms were minimized (2500 minimization steps for both steepest descent and conjugate gradient, for a total of 5000 minimization time); (2) minimization of water and hydrogen atoms, maintaining the solute restrained at 50 kcal/mol force constant, with 20,000 minimization steps (10,000 steepest descent and 10,000 conjugate gradient); (3) side chains of the protein, water, and hydrogen atoms were minimized for 50,000 minimization steps (25,000 with the steepest descent and 25,000 with the conjugate gradient); (4) finally, 100,000 minimization steps of complete minimization (50,000 with the steepest descent and 50,000 with the conjugate gradient) were performed without any restraint. Successively, water molecules, ions, and protein were thermally equilibrated: (1) 5 ns of NVT ensemble with the Langevin thermostat by gradually heating from 0 to 300 K every 50 k by gradually rescaling solute restraints from a force constant of 10 to 0.5 kcal/mol Å2; (2) 5 ns of NPT equilibration at 1 atm with the Berendsen barostat by gradually rescaling restraints from 0.5 to 0.0 kcal/mol Å2; (3) 5 ns of NPT equilibration with no restraints. Trajectories and pre-process data were analysed using the CPPTRAJ module [37], Visual Molecular Dynamics (VMD) graphics vers. 1.93 [38], and MAESTRO GUI. All the conformations visited during the MDs were clustered through the CPPTRAJ module [37] employing hierarchical algorithms, and for the most representative cluster populations, intermolecular interaction energies were analysed via the Molecular Mechanics/Generalized Born Surface Area (MM/GBSA) equation [39]. All images were rendered using Maestro GUI Suite 2021-1 (Schrödinger Release 2021-1) and Adobe Illustrator (Adobe Systems, San Jose, CA, USA).

### 2.11. Alpha Screen

Recombinant human LIFR (His Tag) and biotinylated recombinant human LIF were purchased from Sino Biologicals (Sino Biological Europe GmbH, Dusseldorf, Germany) and R&D Systems (Abingdon, UK), respectively, and both were reconstituted as required by the manufacturer.

Inhibition of LIFR/LIF binding by mifepristone was measured by Alpha Screen (Amplified Luminescent Proximity Homogeneous Assay). The assay was performed in white, low-volume, 384-well AlphaPlates (PerkinElmer, Waltham, MA, USA) using a final volume of 25 μL and an assay buffer containing 25 mM Hepes (pH 7.4), 100 mM NaCl, and 0.005% Kathon. The concentration of DMSO in each well was maintained at 5% vol/vol.

LIFR (His Tag, final concentration 4.5 nM) was incubated with either mifepristone (10 concentrations, from 4.12 nM to 200 µM) or a vehicle for 45 min under continuous shaking. Then, LIF was added (biotinylated, final concentration 20 nM), and the samples were incubated for 15 min prior to adding His-Tag acceptor beads (final concentration 20 ng/µL) for 30 min. Then, streptavidin donor beads were added (final concentration 20 ng/µL), and the plate was incubated in the dark for 3 h and then read in an EnSpire Alpha multimode plate reader (PerkinElmer, Waltham, MA, USA).

### 2.12. Transactivation

To perform STAT3 transactivation, HepG2 (HB, 8065 from ATCC), an immortalized human epatocarcinoma cell line was used. On day 0, HepG2 were seeded at 7.5 × 10^4^ cells/well in a 24-well plate and maintained at 37 °C and 5% CO_2_ in E-MEM supplemented with 10% FBS, 1% glutamine, and 1% penicillin/streptomycin. On day 1, cells were transiently transfected with the reporter plasmid pGL4.47[luc2P/SIE/Hygro] (200 ng) (CAT#: E4041 Promega, Madison, WI, USA), a vector encoding the *h*LIFR (CAT# RC226327) (100 ng) and CD130 (IL6ST) (100 ng) (CAT#: RC215123, OriGene Technologies, Inc. Rockville, MD, USA), and finally a vector encoding the human RENILLA luciferase gene (pGL4.70) (100 ng) (Promega, Madison, WI, USA). On day 2, cells were primed with the cytokine LIF (10 ng/mL) alone or in combination with mifepristone (10, 20, 50 μM). Then, after 24 h, cellular lysates were assayed for luciferase and RENILLA activities using the Dual-Luciferase Reporter assay system (Promega, Madison, WI, USA). Luminescence was measured using a Glomax 20/20 luminometer (Promega, Madison, WI, USA). LUCIFERASE activities (RLU) were normalized with RENILLA activities (RRU).

### 2.13. Flow Cytometry

MIA-PaCa2 cells were seeded in 6-well tissue culture plate (cell density 700 × 10^3^/well) in 100 µL of DMEM medium supplemented with 10% foetal bovine serum, 1% L-glutamine, and 1% penicillin and streptomycin at 37 °C and 5% CO_2_. Cells were serum-starved for 24 h and then incubated with LIF (10 ng/mL) alone or plus mifepristone (10, 20 µM) or a vehicle for 24 h. The intracellular flow cytometry staining for Ki-67 was performed using the following reagents: Ki-67 Monoclonal Antibody (SolA15), Alexa Fluor™ 488, (eBioscience™, San Diego, California, USA) and 7-AAD to characterize the cell cycle phases G0-G1 and S-G2-M. Before intracellular IC-FACS, staining cells were fixed for 30 min in the dark using IC Fixation buffer (eBioscience™) and then permeabilized using Permeabilization buffer (10X) (eBioscience™). The staining for Annexin V was performed using the following reagent: Annexin V Antibody (A13199, Thermofisher Scientific, Waltham, MA, USA) to evaluate the apoptosis rate. Briefly, 5 μL of FITC annexin V was added to each 100 μL of cell suspension, and cells were incubated the at room temperature for 15 min. Flow cytometry analyses were carried out using a 3-laser standard configuration ATTUNE NxT (LIFe Technologies, Carlsbad, CA, USA). Data were analysed using FlowJo software (TreeStar) and the gates set using a fluorescence minus-one (FMO) control strategy. FMO controls are samples that include all conjugated Abs present in the test samples except for one. The channel in which the conjugated Ab is missing is the one for which the fluorescence minus one provides a gating control.

### 2.14. Wound Healing Assay

MIA PaCa-2 cells were seeded in DMEM complete medium at 800 × 10^3^ cells/well into a 24-well plate and were used at a 70–80% confluence rate [40]. On day 1, the cell monolayers were gently scraped vertically with a new 0.2 mL pipette tip across the centre of the well; during the scratch, the medium wasn’t removed to avoid cell death. After scratching, the well was gently washed twice with PBS (Euroclone, Milan, Italy) to remove the detached cells and cell debris, and finally, fresh medium containing LIF (10 ng/mL) alone or in combination with mifepristone (10 µM, 20 µM) or EC359 (25 nM) was added into each well. Immediately after scratch creation, the 24-well plate was placed under a phase-contrast microscope, and the first image of the scratch was acquired (T = 0 h) using a OPTIKAM Pro Cool 5-4083.CL5 camera. Cells were grown for an additional 48 h, and images were taken at 48 h. The gap distance between scrape borders was quantified by assessing that area between the two margins of the scratches. All experiments were performed in triplicate.

### 2.15. Western Blot Analysis

MIA-PaCa 2 cells were seeded in a 6-well tissue culture plate (cell density 1.5 × 10^6^/well) in DMEM medium supplemented with 10% foetal bovine serum, 1% L-glutamine, and 1% penicillin and streptomycin at 37 °C and 5% CO_2_. Cells were serum-starved for 24 h and then incubated with LIF (10 ng/mL) alone or plus mifepristone (10, 20, 50 µM) for 10 min. Total lysates were prepared by homogenization of MIA-PaCa2 cells in Ripa buffer containing phosphatase and protease inhibitors. Protein extracts were electrophoresed on 12% acrylamide Tris-Glycine gel (Invitrogen), blotted to the nitrocellulose membrane, and then incubated overnight with primary Abs against STAT3 (sc-8019 1:500; Santa Cruz Biotechnology) and phosho-Stat3 (GTX118000 1:1000; Genetex). Primary Abs were detected with the HRP-labelled secondary Abs. Proteins were visualized by Immobilon Western Chemiluminescent Reagent (MilliporeSigma) and iBright Imaging Systems (Invitrogen). Quantitative densitometry analysis was performed using ImageJ software. The degree of STAT3 phosphorylation was calculated as the ratio between the densitometry readings of p-STAT3/STAT3.

### 2.16. AmpliSeq Transcriptome

High-quality RNA was extracted from tumour gastric mucosa and healthy mucosa using the PureLink™ RNA Mini Kit (Thermo Fisher Scientific), according to the manufacturer’s instructions. RNA quality and quantity were assessed with the Qubit^®^ RNA HS Assay Kit and a Qubit 3.0 fluorometer followed by agarose gel electrophoresis. Libraries were generated using the Ion AmpliSeq™ Transcriptome Human Gene Expression Core Panel and Chef-Ready Kit (Thermo Fisher Scientific), according the manufacturer’s instructions. Briefly, 10 ng of RNA was reverse transcribed with SuperScript™ Vilo™ cDNA Synthesis Kit (Thermo Fisher Scientific, Waltham, MA, USA) before library preparation on the Ion Chef™ instrument (Thermo Fisher Scientific, Waltham, MA, USA). The resulting cDNA was amplified to prepare barcoded libraries using the Ion Code™ PCR Plate and the Ion AmpliSeq™ Transcriptome Human Gene Expression Core Panel (Thermo Fisher Scientific, Waltham, MA, USA) Chef-Ready Kit, according to the manufacturer’s instructions. Barcoded libraries were combined to a final concentration of 100 pM and were used to prepare Template-Positive Ion Sphere™ (Thermo Fisher Scientific, Waltham, MA, USA) particles to load on Ion 540™ Chips, using the Ion 540™ Kit-Chef (Thermo Fisher Scientific, Waltham, MA, USA). Sequencing was performed on an Ion S5™ Sequencer with Torrent Suite™ Software v6 (Thermo Fisher Scientific). The analyses were performed with a range of fold <−2 and >+2 and a *p* value < 0.05, using Transcriptome Analysis Console Software (version 4.0.2) certified for AmpliSeq analysis (Thermo Fisher). The transcriptomic data were deposited as a dataset in the Mendeley data repository (Mendeley Data, https://doi.org/10.17632/kczfm6pjw2.1).

### 2.17. Statistical Analysis

Statistical analysis was carried out using one-tailed unpaired Student’s *t*-test comparisons (* *p* < 0.05) using the Prism 8.0 software (GraphPad, San Diego, CA, USA).

## 3. Results

### 3.1. LIF and LIFR Expression in PDAC

**Figure 1:**
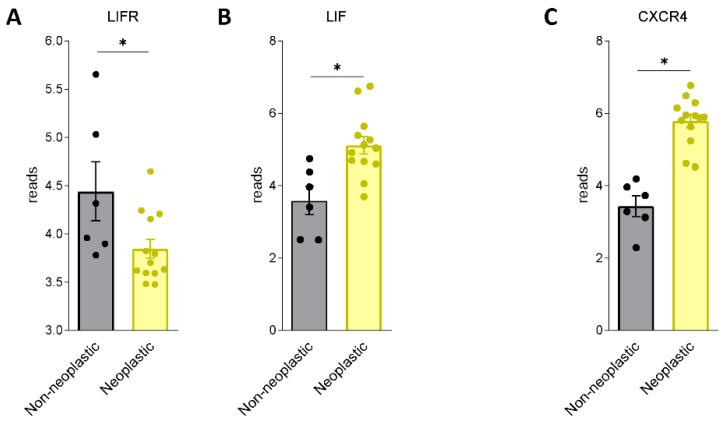
LIF and LIFR show an opposite regulation in human PDAC. RNA-seq analysis of non-neoplastic and neoplastic mucosa of PDAC from GSE196009 repository. Each dot represents a patient. Data shown represent the gene profile expression of (**A**) LIFR, (**B**) LIF, chemokine receptor type 4 (**C**) CXCR4. Results are the mean ± SEM of 6 (Non-neoplastic) and 13 (Neoplastic) samples per group. * *p* < 0.05.

We first assessed whether LIF and LIFR are expressed in human PDAC. For this purpose, we used a human repository of PDAC tissues. The repository includes cancer tissues along with the adjacent normal pancreatic tissue from 12 Japanese patients (Repository GSE196009 series) (Figure 1A,B). The results of these studies demonstrate that while LIF and LIFR are expressed in non-neoplastic pancreases, the two genes are differently regulated in the cancer tissues: thus, while LIFR is downregulated in PADC cancer in comparison with the adjacent tissue, LIF undergoes an opposite modulation.

**Figure 2:**
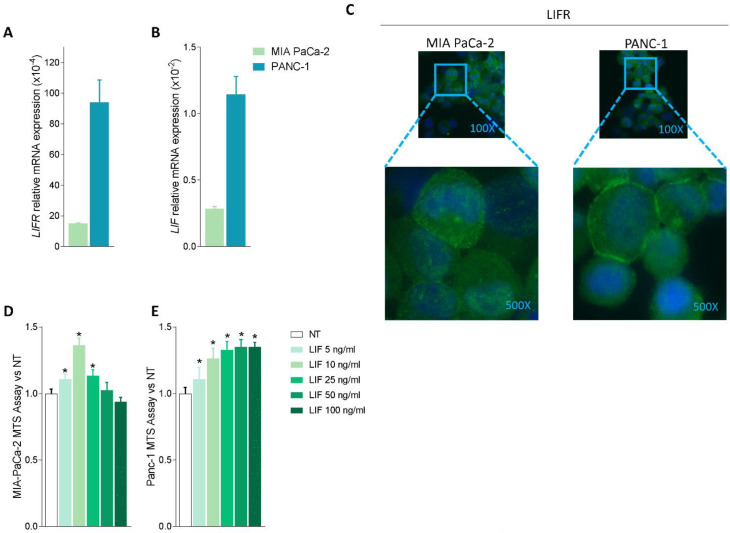
LIF/LIFR expression is modulated in MIA PaCa-2 and PANC-1 cells and LIF induces cell proliferation. Relative mRNA expression of (**A**) LIFR and (**B**) LIF in MIA PaCa-2 (green) and PANC-1 (light blue) cell lines. (**C**) Immunofluorescence analysis of LIFR expression in PANC-1 and MIA PaCa-2 cell lines (magnification 100× and 500×). Dose-response curve of LIF (5, 10, 25, 50, 100 ng/mL) determined using MTS assay on (**D**) MIA PaCa-2 and (**E**) PANC-1 cell lines. Each value is expressed relative to the non-treated (NT) value, which is arbitrarily set to 1. Results are the mean ± SEM of 10 samples per group. * *p* < 0.05.

Because these data suggest that the LIF/LIFR pathway is modulated in PDAC tissues, we then investigated whether LIF/LIFR expression undergoes the same regulation in two pancreatic cancer lines, MIA PaCa-2 and PANC-1, which are widely used as in vitro models for PDAC [41]. The two cell lines presented a substantial difference in the expression of the LIF/LIFR pathway, which was significantly less expressed in MIA PaCa-2 cells in comparison to PANC-1, as demonstrated by mRNA expression profiles (Figure 2A,B) and immunofluorescence analysis (Figure 2C).

However, despite this difference, both cell lines exhibit a concentration-dependent proliferation when challenged with LIF (5, 10, 25, 50, and 100 ng/mL); however, while the MIA PaCa-2 cells showed a biphasic response (Figure 2D), PANC1 cells exhibited a linear progression of cell proliferation (measured by the MTS assay) in response to increasing concentrations of LIF (Figure 2E). The fact that exposure of MIA PaCa-2 cells to higher concentrations of LIF (25, 50, and 100 ng/mL) promoted a reduction of cell vitality is consistent with the finding that, in some systems, this cytokine exerts direct cytotoxic effects (Figure 2D) as reported by us and others in gastric cancer cell lines [16,42]. Because the effects exerted by low concentrations of LIF (5–10 ng/mL) were similar in the two cell lines, we used MIA PaCA-2 for the following experiments.

**Figure 3:**
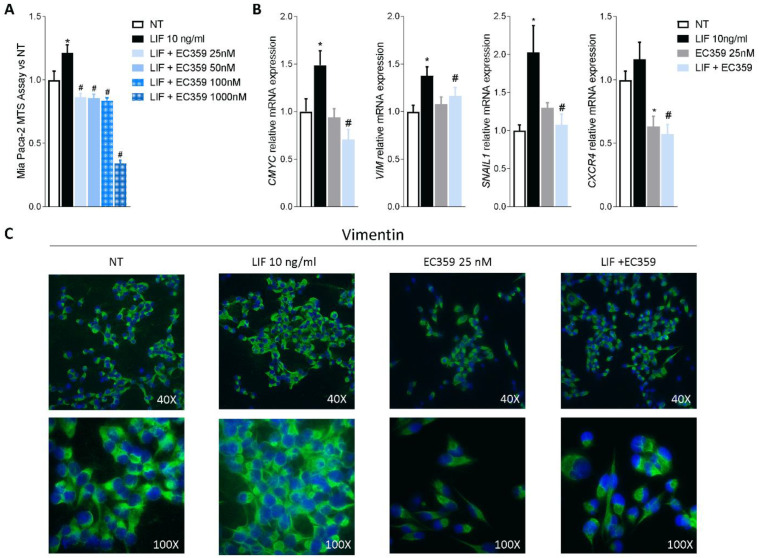
LIFR inhibition reverses pancreatic cancer cell proliferation and EMT process promoted by LIF. MIA PaCa-2 cells were serum-starved and primed with LIF (10 ng/mL) alone or in combination with increasing concentrations of LIFR antagonist, EC359 (25, 50,100, 1000 nM). Data shown are (**A**) dose-response curve of EC359 (25, 50, 100, 1000 nM) determined using MTS assay on cells. Each value is expressed relative to the non-treated (NT) value, which is arbitrarily set to 1. Results are the mean ± SEM of 10 samples per group. (**B**) Relative mRNA expression of the proliferation marker *C-Myc*; the EMT markers *VIM* and *SNAIL-*1; and *CXCR4*. Each value is normalized to *GAPDH* and is expressed relative to those of positive controls, which are arbitrarily set to 1. Results are the mean ± SEM of five samples per group (* represents statistical significance versus NT, and # versus LIF, *p* < 0.05). Panel (**C**) shows changes in vimentin expression assessed by immunofluorescence analysis in MIA PaCa-2 cells triggered with LIF (10 ng/mL) alone or in combination with EC359 25 nM.

Since previous studies have shown that challenging cancer cells with LIF promotes their proliferation and the epithelial-to-mesenchymal transition (EMT), we functionally assessed whether LIFR inhibition reverses this pattern. For this purpose, MIA PaCA-2 cells were challenged with LIF, 10 ng/mL, alone or in combination with EC359, a selective steroidal LIFR antagonist [23]. As shown in Figure 3A, EC359 (25, 50, 100, and 1000 nM) in a concentration-dependent manner reversed the effects induced by LIF. These effects were statistically significant already at the lowest concentration of EC359 tested (i.e., 25 nM), while EC359 was cytotoxic at 1000 nM. Similarly, the mRNA expression of CMYC, a marker of cell proliferation, was statistically reduced at 25 nM EC359 (Figure 3B). EC359 also reduced the expression of the EMT’s biomarkers Vimentin and Snail Family Transcriptional Repressor 1 (SNAIL-1) along with the expression of Chemokine receptor type 4 (CXCR4), a biomarker of PDAC aggressiveness that was also upregulated in the PADC tissues (Figure 3B) [43]. Vimentin is required for cell transition from the EMT state [44], and as shown in Figure 3B, LIFR inhibition decreased both the genes as well as the assembly of vimentin filaments [Figure 3B] that are essential for cytoskeleton reorganization, cell spreading, and migration.

Together, these data demonstrate that LIFR inhibition reverses pancreatic cancer cell proliferation and EMT promoted by LIF/LIFR signalling.

### 3.2. Mifepristone Putative Binding at the LIFR-LIF Interface

**Figure 4:**
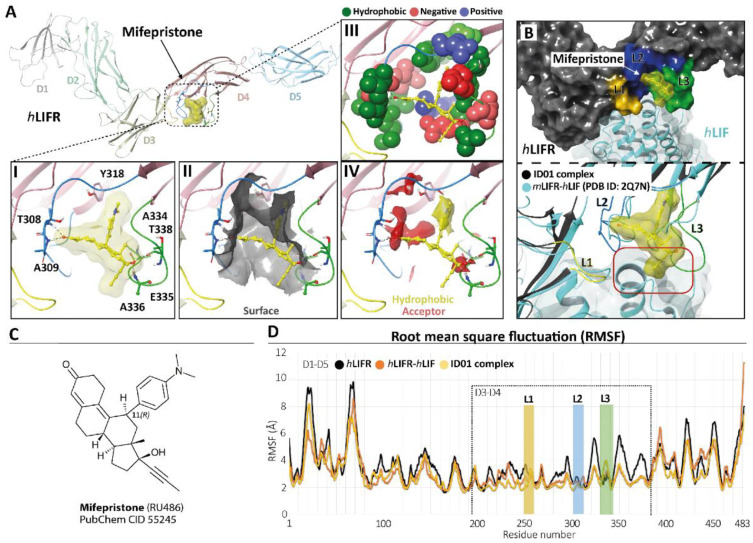
Modelling. (**A**) *h*LIFR-ID01 complex. The protein backbone is displayed in ribbon and coloured by distinguishing the five domains (D1–D5). In the zoom view, (I) the ID01 binding mode of mifepristone (displayed as yellow ball and stick), highlighting the “T-inverted” shape; (II) the “T-inverted” shape well-fit with *h*LIFR surface; (III) the main residues involved in the interactions with the mifepristone; (IV) the hydrophobic (yellow) and hydrogen bond acceptor (red) maps fit with mifepristone. The principal residues are labelled and highlighted in wireframe or CPK(A3), while hydrogen bonds are in dashed black lines. (**B**) Superimposition between the ID01 best MM/GBSA value pose (black) and the *m*LIFR-*h*LIF X-ray (PDB ID: 2Q7N) (cyan). The three loops L1 (255-VSASSG-260), L2 (303-NPGRVTALVGPRAT-316), and L3 (332-KRAEAPTNES-341) are highlighted in yellow, blue, and green, respectively. The red rectangle highlights the clash with the propyne moiety of mifepristone and *h*LIF. (**C**) Two-dimensional structures of mifepristone. (**D**) The root means square fluctuation (RMSF) plot of the three compared systems (ID01 complex, *h*LIFR-*h*LIF, and *h*LIFR) during 100 ns of MDs.

To identify potential LIFR inhibitors, we performed a similarity screening against the FDA-approved drug database using the chemical structure of EC359 [23] as the query. The most similar compound detected by this search was mifepristone [24] (Supplementary Figure S3, panel C), a synthetic steroid and FDA-approved drug with a *p*-(dimethylamino)phenyl group in the 11β-position acting as a progesterone receptor (PR) antagonist (Figure 4, panel C) and clinically used for the induction of medical abortion.

**Figure 5:**
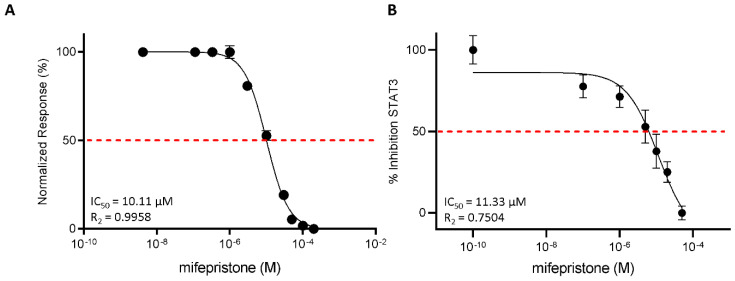
Mifepristone antagonizes LIFR and reduces STAT3 activation. (**A**) Mifepristone inhibition activity of LIFR/LIF binding accessed by a cell-free AlphaScreen assay. (**B**) STAT3 transactivation on HepG2 cells. Results are expressed as mean ± SEM of five samples per group.

Therefore, we investigated whether this agent was able to bind the *h*LIFR through a two-step docking procedure followed by molecular dynamics simulations (MDs) (Supplementary Figure S4). As a first step, we searched for putative binding pockets of the *h*LIFR structure by using the SiteMap (SiteMap, S., LLC, New York, NY, USA, 2021 rip) tool. The method defines both surface cavities and pharmacophore molecular interaction fields, summarized in hydrophobic (yellow), hydrogen bond donor (blue), and/or acceptor (red) areas. The SiteMap analysis yielded five possible binding pockets (named S1 to S5), with only one located between the D3–D4 domains (site S4), directly involved in LIF binding (Supplementary Figure S3, panel B). The pocket was defined by three loops, namely L1 (255-VSASSG-260), L2 (303-NPGRVTALVGPRAT-316), and L3 (332-KRAEAPTNES-341), which were already characterized as binding sites for EC359 [23]. According to the X-ray structure of *h*LIF/*m*LIFR (PDB 2Q7N [27]), loops L1 and L2 are directly involved in the binding of the *h*LIF (Supplementary Figure S3, panel A,B). To study the potential binding mode of mifepristone to *h*LIFR, we performed two-step docking experiments on a box set on the centre of mass of the SiteMap pocket (Supplementary Figure S3, panel B) by using *QM-Polarized Ligands Docking* (QPLD) (Glide, S., LLC, New York, NY, 2021; Jaguar, S., LLC, New York, NY, USA, 2021; QSite, S., LLC, New York, NY, USA, 2021). The best results were then refined by *Induced Fit Docking* (IFD) (Glide, S., LLC, New York, NY, 2021; Prime, S., LLC, New York, NY, USA, 2021) calculations. According to the QPLD G-Score (Supplementary Table S2) and to the match the ligand chemical features with the pharmacophore molecular interaction fields of the pocket calculated by the SiteMap tool (Supplementary Figures S4 and S5, panels A), two QPLD docking poses (ID01 and ID02) were selected and submitted to IFD docking refinements (Supplementary Figure S5, panel B). The IFD results were very conservative in the case of ID01, showing a slight rearrangement of the loops around the ligand. Conversely, IFD refining of ID02 yielded two main families of results (ID02-A and ID02-B; Supplementary Figure S5, panel B), which were both very different compared to the starting point. Interestingly, the ID02-B pose was very similar to ID01 (RMSD 1.22 Å). In light of such considerations, the three different poses of mifepristone, i.e., ID01, ID02-A, and ID02-B, were submitted to 100 ns of MD simulation. The time evolution plot of the ligand root means square deviation (L-RMSD) showed that, after 40 ns, the ID02-A binding mode became unstable (Supplementary Figure S4, panel B), until losing all contact with the receptor. In contrast, the L-RMSD of ID01 and ID02-B showed a stable configuration during the MD. It is noteworthy that ID01 and ID02-B trajectories converged to a very similar binding mode, with mifepristone engaging a very similar pattern of interactions, with the 3-keto group on ring-A oriented toward the loop L2 establishing hydrogen bond (HB) contacts with T308 and/or A309 residues (Supplementary Figure S4, panel C). The D-ring, with the hydroxyl group at the C-17 position, contacted the loop L3, establishing HB with T338 and/or A336 (Supplementary Figure S4, panel C). Nevertheless, clusterization of the trajectories yielded different clusters (Supplementary Table S3), differing for the conformation of the highly flexible loops L1–L3, and in particular loop L3. To identify a representative structure of mifepristone binding *h*LIFR, we therefore evaluated the free energy of the most represented clusters for both simulations through the estimation of the MM/GBSA *ΔG^tot^* value. The most populated cluster from the MD of the complex ID01 was energetically favoured, having the best MM/GBSA Δ*G^tot^* value (−40 Kcal/Mol; Supplementary Table S3), and it was therefore identified as the most representative in the binding mode. As shown in Figure 4, panel A, in the ID01 complex, mifepristone interposes between loops 1 and 2 (L1–L2) (Figure 4, panel A1) by orienting the 3-keto group towards T308 and A309 residues and the propynyl group at 17-position on the steroid scaffold to the space normally occupied by LIF. In this pose, mifepristone fit well into the pocket (Figure 4, panel A2), mainly rich in hydrophobic residues (V307, T308, A309, L310, V311, P313, A315, T316, Y318, L331, A334, A336, P337, T338, and Y342), which contribute to the stabilization of the ligand via Van der Waals contacts (Figure 4, panel A3) as also revealed by the matching of both hydrophobic and acceptor predicted areas (regions favourable for occupancy by hydrophobic and donor groups, respectively). The steroidal scaffold with the *p*-(dimethylamino) phenyl group at the 11β-position was well embedded in the hydrophobic region (yellow) while the 3-keto group fit into the acceptor region (red), establishing two hydrogen bonds (Figure 4, panel A4). By superimposing the ID01 complex on the X-ray LIF-*h*LIF (PDB ID: 2Q7N [27]), we observed that the propynyl moiety clashes with *h*LIF (Figure 4, panel B). This observation could explain the intrinsic activity of mifepristone in inhibiting *h*LIFR activity for competing interaction. Moreover, to further investigate the effect of mifepristone binding to *h*LIFR, we also performed MD simulations of the *h*LIFR apo structure, and 100 ns of the *h*LIF/*h*LIFR complex. The 3D model structure of the *h*LIF/*h*LIFR complex was built as described in the Materials and Methods. Interestingly, the comparison of RMSD plots calculated on the backbone of the overall structure, including D1–D5 domains, of the *h*LIFR apo structure with the *h*LIF/*h*LIFR complex showed higher values for the complex with respect to the apo structure, albeit the RMSD calculated on domains D3 and D4 were comparable (Supplementary Figure S6). The comparison of the residue root means square fluctuations (RMSF) of the three systems ID01, *h*LIFR-*h*LIF, and *h*LIFR (Figure 5D) showed that, as expected, the region involved in the binding with mifepristone (domain D3–D4 and L1, L2, L3 loops) was generally less fluctuating with respect to the *h*LIFR apo structure and comparable to the *h*LIFR-*h*LIF profile. Moreover, the binding of the agonist and of the antagonist markedly affected the D4 domain intrinsic dynamic, reducing the fluctuations of loops of β-sheets, including those not involved in the binding (residues P285-L295; L320-Y328; I354-N360). Compared to the agonist LIF, mifepristone binding to *h*LIFR slightly reduced the dynamics of the D3 domain and of the loop L3.

Taken together, these docking and MDs results suggest that mifepristone binds loops L2 and L3, acting as a competitive antagonist of LIF.

### 3.3. Functional Characterization of Mifepristone as LIFR Antagonist by Alpha Screen and Transactivation Assay

To strengthen the docking results, we then investigated the efficacy of mifepristone as an LIFR antagonist in a cell-free system using the AlphaScreen assay (Figure 5A). The results of these studies demonstrated that mifepristone inhibits LIF binding to LIFR in a concentration-dependent manner with an IC50 of ≈10 µM. These AlphaScreen results were then confirmed by transactivation assay performed in HepG2 cells, a liver cancer cell line, transiently co-transfected with the following vectors: hLIFR, hgp13,0 and pGL4.47[luc2P/SIE/Hygro] containing the gene for Luciferase under the control of the STAT3 inducible elements (SIE), and finally a vector for human Renilla gene as a control for transfection efficiency and cell viability. As shown in Supplementary Figure S2, the LIF binding to LIFR induces the assembly of a LIFR/gp130 heterodimer and activates downstream signalling, including the endogenous Janus kinase2 (JAK2), which becomes activated and phosphorylates the cytoplasmic domains of both receptors. These phosphorylations promote the recruitment and phosphorylation of the signal transducer and activator of transcription 3 (STAT3), which in turn dimerizes and translocates to the nucleus, binding to the transfected SIE and activating the transcription of the luciferase gene. These events lead to signal emission. Mifepristone reduced STAT3 activation in a concentration-dependent manner with an IC50 of ≈11 µM (Figure 5B).

### 3.4. Effect of Mifepristone on PDAC Cell Proliferation and EMT

**Figure 6:**
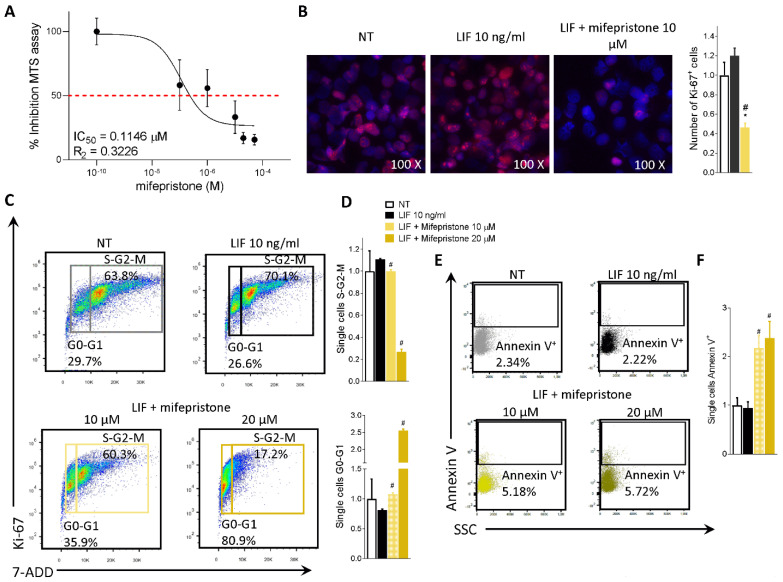
Mifepristone reduces MIA PaCa-2 cell proliferation and reverts EMT process. (**A**) Dose-response curve of mifepristone (0.1, 1, 10, 20, 50 µM) was determined using MTS assay on MIA PaCa-2 cells (n = 10). (**B**) Immunofluorescence analysis of Ki-67 positive MIA PaCa-2 cells left untreated or challenged with LIF (10 ng/mL) alone or in combination with mifepristone 10 µM; and estimated number of Ki-67 positive cells. MIA PaCa-2 cells were serum-starved and challenged with a vehicle or LIF 10 ng/mL alone or in combination with mifepristone (10, 20 µM) for 24 h. Cell cycle phase analysis was performed by Ki-67/7-AAD staining through IC-FACS. (**C**) Representative IC-FACS shows cell cycle fraction in each experimental group. (**D**) Data shown are frequencies of cells in the G0-G1 phase and S-G2-M phase. (**E**) Representative IC-FACS shows cell cycle fraction in each experimental group. (**F**) Data shown are frequencies of Annexin V^+^ single cells. Each value is normalized to untreated cells, expressed relative to those of controls, which are arbitrarily set to 1. Results are the mean ± SEM of three samples for group (* represents statistical significance versus NT, and # versus LIF, *p* < 0.05).

To functionally characterize the effect of mifepristone on MIA PaCa-2 cells, we ran an MTS assay. The MIA PaCa-2 cells were grown in a serum-free medium containing 10 ng/mL LIF alone or in combination with increasing concentrations of mifepristone (0.1, 1, 10, 20, 50 µM) for 48 h. As shown in Figure 6A, mifepristone reversed the LIF-proliferative effect in a concentration-dependent manner with an IC50 of 0.11 µM.

The action of mifepristone on cell replication was also investigated by immunofluorescence analysis of Ki-67. As shown in Figure 6B, while LIF increased the number of Ki-67 positive cells, this pattern was reversed by LIFR inhibition with mifepristone (Figure 6B).

In addition, challenging MIA PaCa-2 cells with mifepristone modulated the cell cycle progression by Ki-67/7-AAD IC-FACS staining (Figure 6C,D) and apoptosis cell rates accessed by Annexin V staining (Figure 6E,F). As mentioned above MIA PaCa-2 cells (Figure 2B) and PDAC tissue (Figure 1B) express LIF that acts as an autocrine factor to promote cancer cell proliferation [45]. Building on these molecular features, we carried out a cell cycle analysis on MIA PaCa-2 cells challenged with LIF and mifepristone. The results of these investigations demonstrated that while LIF increases the S-G2-M transition, this finding was reversed by mifepristone, which blocks the transition from resting G0-G1 to the S-G2-M cell cycle phase (Figure 6D) and increases the apoptosis cell rates measured as frequencies of Annexin V^+^ single cells (Figure 6F), in a statistically significant manner (mifepristone 10, 20 µM) (*p* < 0.05).

**Figure 7:**
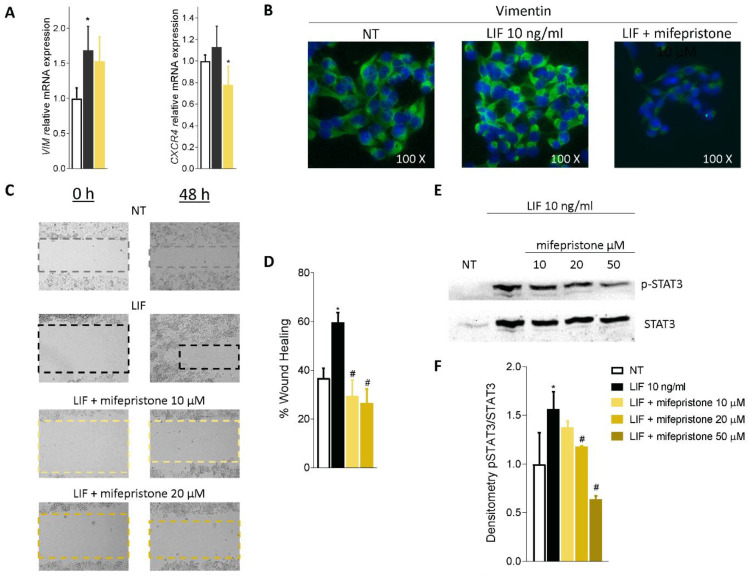
Mifepristone inhibits in vitro migration in STAT3-dependent signalling. (**A**) Relative mRNA expression of Vimentin and CXCR4. (**B**) Immunofluorescence analysis of Vimentin expression. (**C**) Scratch wound healing assay. MIA PaCa-2 cell monolayers were scraped in a straight line using a p200 pipette tip; then, they were left untreated or primed with LIF 10 ng/mL alone or in combination with mifepristone 10 or 20 µM and EC359 25 nM. The wound generated was captured at 0 and 48 h of incubation with the compounds above described. The images show cell migration at the two times point indicated. (**D**) Images of obtained points were analysed, measuring scraped area and its closure vs. the first time point at 0 h. Results are the mean ± SEM of three samples per group (* represents statistical significance versus NT, and # versus LIF, *p* < 0.05). (**E**) Analysis of STAT3 signalling pathway. Representative Western blot analysis of STAT3 and phospho-STAT3, proteins in MIA-PaCa-2 cells exposed to LIF (10 nM) alone or in combination with increasing concentration of mifepristone (10, 20, 50 µM) for 20 min. (**F**) Densitometric analysis demonstrating phospho-STAT3/STAT3 ratio.

Mifepristone also reversed EMT features in MIA PaCa-2 cells challenged with LIF, as shown in Figure 7A. Thus, mifepristone downregulated vimentin mRNA expression and reduced the assembly of vimentin fibres, as displayed in Figure 7B.

Because these findings suggest that exposure to LIF might promote the acquisition of a migratory phenotype, we measured the motility of MIA PaCA-2 cells in scratch wound healing assay, as described in the Materials and Methods (Figure 7C). For these purposes, MIA PaCa-2 cells were grown in a complete serum-starved DMEM medium, and after the production of a scratch (Day 0), cells were challenged with 10 ng/mL LIF, alone or in combination with mifepristone (10 and 20 µM). The ability of cells to gain a migratory phenotype was calculated by the measuring of the area between the two scratch borders at the time points 0 h and 48 h. As illustrated in Figure 7D, LIF promoted cell migration and wound closure, with a reduction of the wound area by 59.69% at 48 h. These findings were reversed by treatment with mifepristone (*p* < 0.05). Mifepristone significantly reduced MIA PaCa-2 migration in a concentration-dependent-manner.

We then investigated whether LIF/LIFR modulates JAK and STAT3 phosphorylation and found that while LIF 10 ng/mL increased the phosphorylation of STAT3, this effect was reversed by mifepristone in a concentration-dependent manner, with a maximal effect at 50 µM (Figure 7E,F).

Altogether, the data presented herein suggest that mifepristone functions as LIF/LIFR antagonist in PDAC cell lines.

### 3.5. RNAseq Analysis of the Effects of LIF and Mifepristone on MIA PaCa-2 Cells

**Figure 8:**
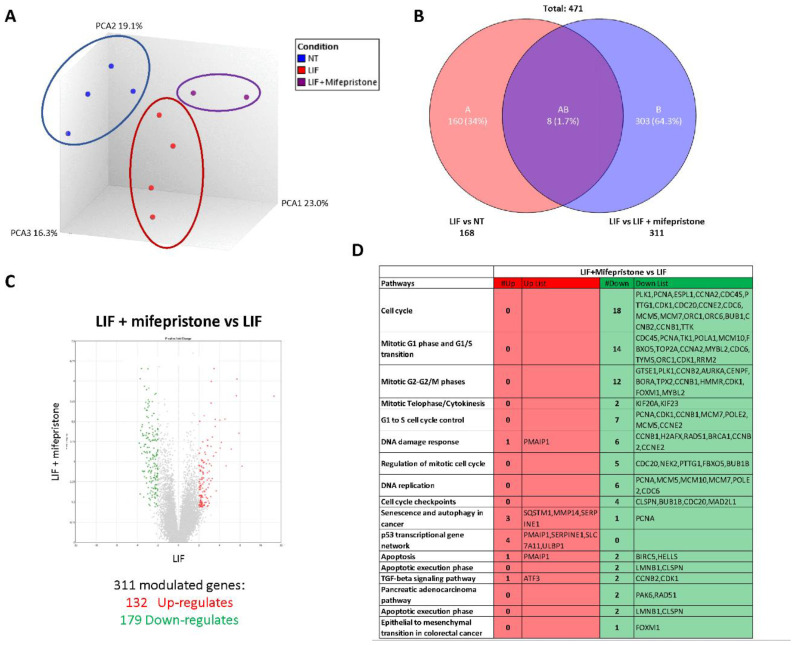
Analysis of mifepristone effects on MIA PaCa-2 cells challenged with LIF by RNAseq. (**A**) Heterogeneity characterization of the three experimental groups as shown by principal component analysis (PCA) plot. (**B**) Venn diagram of differentially expressed genes showing the overlapping region between the three experimental groups. (**C**) Volcano plots of transcripts differentially expressed between different experimental groups (fold change <−2 or >+2, *p* value < 0.05). Red dots represent significantly upregulated genes, and green dots represent significantly downregulated genes. (**D**) Table showing genes modulated by LIF/mifepristone versus LIF.

To gain further detail on the transcriptional profile promoted by LIF and mifepristone, AmpliSeq Transcriptome analysis (RNAseq) was performed on MIA PaCa-2 cells left untreated or treated with LIF alone or in combination with 10 µM mifepristone for 24 h. The Principal Component Analysis (PCA) of the transcriptome (Figure 8A) revealed major dissimilarities between MIA PaCa-2 left untreated or treated with LIF or LIF/mifepristone.

The Venn diagram analysis of differentially expressed transcripts, shown in Figure 8B, allowed the identification of 471 transcripts that were differentially regulated across the three experimental groups: 168 transcripts were differentially modulated by LIF versus untreated cells (Subset A), while 311 transcripts were differentially modulated by exposure to LIF/mifepristone in comparison to LIF alone (Subset B); the AB subset includes only eight transcripts that were modulated by LIF and LIF/mifepristone in comparison to untreated cells. The volcano plot illustrated in Figure 8C identifies transcripts differentially expressed between cancer cells challenged with LIF alone and LIF plus mifepristone. Specifically, the analysis shows that of 311 t genes, 132 were upregulated and 179 downregulated (Figure 8C). The *per pathways* analysis of these differentially expressed gene sets performed by the TAC software (Affymetrix) to dissect the molecular pathways underlines the effects of mifepristone, demonstrating that the largest families of downregulated genes belong to cell cycle signalling, mitotic G1 phase and G1/S transition, mitotic S-G2/M phases, DNA damage response, regulation of mitotic cell cycle, cell cycle checkpoint, and upregulated genes belonging to the p53 transcriptional gene network (Figure 8D). Particularly, the most downregulated gene was *Ribonucleotide Reductase Regulatory Subunit M2 (*RRM2), with a Fold Change (FC) of –3.64. RRM2 was upregulated in PDAC, and its expression was correlated with gemcitabine resistance [26]. Additionally, mifepristone downregulated the expression of genes codifying for various cyclins, including the cyclin A2 (CCNA2) (FC: −3.56), cyclin B1 (CCNB1) (FC: −2.96), cyclin B2 (CCNB2) (FC: −2.96), and cyclin-dependent kinase 1 (CDK1) (FC: −2.71), whose expression is significantly associated with poor prognosis [46]. In contrast, the expression of few pro-apoptotic genes was downregulated by mifepristone: *Baculoviral IAP Repeat Containing 5* (BIRC5) (FC: 3.12) and *Helicase, Lymphoid Specific* (HELLS) (FC: −2.13).

On the other hand, the most upregulated gene by mifepristone was SLC7A11, also known as xCT, a cysteine transporter involved in the inhibition of the ferroptosis process, an intracellular iron-dependent programmed cell death [47]. Among the other highly modulated genes were the *Phorbol-12-Myristate-13-Acetate-Induced Protein 1* (PMAIP1), a tumour suppressor gene frequently downregulated in PDAC, which was robustly modulated (FC: +2.28) [38]. PMAIP1 belongs to the p53 network and is critical for caspase activation and apoptosis promotion (Figure 8D).

## 4. Discussion

LIF is a secretory glycoprotein endowed with multiple biological functions, including embryonic stem cell self-renewal, embryonic implantation and placental formation, and stimulation or inhibition of cell proliferation and differentiation. LIF is ectopically overexpressed in multiple solid tumours, exerting promoting activity in tumour growth, metastasis formation, and chemotherapy resistance [45,48,49]. The *LIF* gene is highly conserved across species and in humans localizes to a 76 kb segment on chromosome 22q12.1–12.2; its transcription is modulated by various transcription factors, including Transforming Growth Factor beta (TGF-β) and leptin [45,48,49]. In PDAC cells, the LIF/TGFβ pathway promotes the epithelial mesenchymal transition and tumour aggressiveness and is regarded as a promising therapeutic target [50]. LIF signalling in cancer cells is mediated by its binding to the LIFR complex and JAK/STA3 phosphorylation [51]. Several solid tumours exhibit an aberrant upregulation of JAK/STAT3 signalling via LIF autocrine or paracrine production [45,52] and, as shown in the present study, the LIF/LIFR pathway is represented in PDAC (Figure 1). These findings are consistent with the observation that, in comparison to non-neoplastic subjects, circulating levels of LIF are significantly increased in PDAC patients and correlate with poor prognosis and cancer aggressiveness [53,54].

By challenging PC cell lines such as MIA PaCa-2 and PANC-1 with LIF, we observe an incremental proliferation rate with the acquisition of the molecular signature of EMT. The small steroidal molecule LIFR inhibitor, EC359, reversed these changes as well as the downregulation of VIM and Snail1 expression, validating the pro-oncogenic potential of LIF/LIFR in PC cell lines.

Based on the steroidal structure of EC359, we performed a similarity screening of the FDA-approved drug database. Our efforts allowed the identification of mifepristone as a potential LIFR antagonist. Mifepristone is clinically used for the induction of medical abortion but has been tested in clinical trials for its anti-proliferative potential [24].

Using docking and MD studies, we have demonstrated that mifepristone binds the receptor complex of LIFR interacting with the D3–D4 domain and L1–L2 loops, orienting this 17-propyne group towards the pocket normally occupied by LIF. This receptor’s occupancy pose supports the view that mifepristone interferes with the agonist binding of LIF to LIFR, preventing LIFR activation, as confirmed by the AlphaScreen assay.

Upon binding to the LIFR/gp130 complex, LIF activates multiple signalling, including STAT3, AKT, MAPK, and mTOR [19,51,55]. Importantly, while LIFR does not have intrinsic tyrosine kinase activity, both LIFR and gp130 constitutively associate with the JAK-Tyk family of cytoplasmic tyrosine kinases; thus, when LIF binds to the LIFR complex, it leads to activation of the JAK/STAT pathway [51]. Consistent with these earlier findings, we have shown that co-treating MIA PaCa-2 cells with mifepristone reverses the LIF proliferative effect mediated by LIF in a concentration-dependent manner, reducing cell vitality and the number of ki-67+ cells, but also diminishes STAT3 phosphorylation induced by LIF, as well as regulation of vimentin and migratory phenotype acquisition of the cell line, further confirming its value in the pro-oncogenic LIF/LIFR axis inhibition.

To gain further insights into the mode of action of mifepristone on LIF/LIFR signalling, we carried out a RNAseq analysis on MIA PaCa-2 cells challenged with LIF. The results of these studies demonstrated that LIFR antagonism decreases the rate of cell proliferation by multiple mechanisms, including the regulation of 14 genes involved in the G1-S phase transition and 12 genes involved in the G2/M shift. The most downregulated of these genes was RRM2, which is associated with a poor prognosis in pancreatic [26] and lung [56] cancers. The level of expression of RRM2 is a validated biomarker of sensitivity of PDAC to chemotherapy, and high levels of expression predict poor prognosis and resistance to gemcitabine [57,58].

In addition to RRM2, several cyclins were modulated by exposure of Mia PaCa-2 to LIF, including CCNA2 and CCNB1. CCNA2 [59], a widely expressed cyclin that regulates the G1-to-S and G2-to-M cell cycle transition, is aberrantly overexpressed in PDAC (Supplementary Figure S1), and higher levels of expression correlate with poor prognosis and chemoresistance [46]. The CCNB1 has an essential role in assembling a complex with CDK1, which in turn promotes the transition from the G2 phase to mitosis [60]. Similarly to CCNA2, overexpression of CCNB1 and CDK1 has been detected in several human cancers, including PDAC [61]. One important result of the present study was the demonstration that exposure to MiaPaCA-2 cells to mifepristone reverses the regulation of these cyclins, providing robust evidence that regulation of the LIF-LIFR pathway might have mechanistic relevance for PDAC progression, further strengthening the potential clinical utility of LIFR antagonism in PDAC patients.

Additionally, exposure to mifepristone downregulated the expression of several apoptotic genes, including BIRC5 and HELLS, while increased the expression of SLC7A11, a transporter involved in ferroptosis, an iron-dependent cell death that has been shown to be regulated in PDAC and other cancers [47]. Together, these data suggest that mifepristone slows down the progression of PDAC cells though the cell cycle, freezing cells in the G0–G1 phases and retarding the progression toward S-G2-M.

In conclusion, we identified a LIF/LIFR pathway that promotes/maintains oncogenicity in PDAC cell lines. Using docking and pharmacological experiments, we have shown that mifepristone, a clinically approved anti-steroidal agent, functions as a LIFR antagonist, directly binding the LIFR complex and preventing its activation in PDAC cell lines. Present results support the repositioning of mifepristone in the treatment of LIFR expressing PDAC.

## Data Availability

The data presented in this study are available on request from the corresponding authors.

## Supplementary Materials

The following supporting information can be downloaded at: https://www.mdpi.com/article/10.3390/cells11213482/s1. Table S1: Protein-protein contacts between the principal residues located on the *h*LIF loops and both *m/h*LIFr-LIF complex; Table S2: QPLD and IFD score values; Table S3: MM/GBSA values of the two most populated clusters, Figure S1: RNA-seq analysis of non-neoplastic and neoplastic mucosa of PDAC from GSE196009 repository. Each dot represents a patient. Data shown represent the gene profile expression of the cell cycle check point protein RRM2, CCNA2, CCNB1 and CDK1 (* *p* < 0.05); Figure S2: Strategy of transactivation of STAT3 used to investigate the role of mifepristone as LIFR antagonist. HepG2 were transiently transfected with hLIFR, hgp130 and pGL4.47[luc2P/SIE/Hygro] pGL4.47[luc2P/SIE/Hygro] and a vector for human Renilla gene. Cells were primed with LIF 10 ng/ml alone or in combination with Mifepristone (10, 20, 50 µM). Luciferase activity served as a measure of the transcription of luciferase after the binding of pSTAT3 to Stat3 inducible elements (SIE) following activation and heterodimerization of LIFR: Gp130 LIF-induced (on left). Instead, Mifepristone can inhibit LIFR: LIF binding and blockade the pathway downstream with a decrease of luciferase signal (on right); Figure S3: (A) Superimposition of hLIFR (black) on the mLIFR–hLIF (cyan) complex and the zoom-view of the most important interactions between the two structures. In the front and back view, the h/mLIFR residues are represented in stick, while those on hLIF are visualized in ball and stick and coloured in green. The RMSD value was calculate on the backbone. (B) SiteMap surface (gray), hydrophobic, donor, and acceptor predicted maps coloured in yellow, blue, and red, respectively. Centroid and coordinates (XYZ) calculated on the site points (white) useful to set up the box for docking studies; Figure S4: (A) Schematic representation of the three mains in silico steps: 1. QPLD, 2. IFD, and 3. MD. (B) The ligand root means square deviation (L-RMSD) and (C) the Protein-Ligand (PL) contacts (hydrogen bonds, HB) plots of ID01 (yellow), ID02-A (red), and ID02-B (green) complexes, throughout 100 ns of MDs. Values are expressed as a percentage of interaction between ligand and the principal residues of the protein during the trajectories. Figure S5: Depiction of the (A) QPLD and (B) IFD docking poses for each complex (ID01, yellow; ID02-A, red; and ID02-B, green). The principal residues and ligands are labelled and shown in stick and ball and stick, while hydrogen bonds are pictured as dashed black lines. Both pocket and ligand surfaces, and the pharmacophore molecular interaction fields of the pocket calculated by SiteMap, are highlighted. The hydrophobic, hydrogen bond donor and/or acceptor areas are coloured in yellow, blue and red, respectively; Figure S6: Comparison of the root mean square deviation (RMSD) plots of the MD of the apo structure (black) with the respect to the hLIF/hLIFR complex (orange) for D1-D5 (left) and D3-D4 (right) domains; Figure S7: MIA PaCa-2 cells were serum starved and challenged with vehicle or LIF (10 ng/ml) alone or in combination with mifepristone (10 µM) for 24 h. The map shows the pathway main regulated by mifepristone action. The upregulated and genes (Fold Change < −2 or > 2, *p* value < 0.05) are represented in the map in green.
