# Supplementary material for: Repositioning Mifepristone as a Leukaemia Inhibitory Factor Receptor Antagonist for the Treatment of Pancreatic Adenocarcinoma
Source: Cells. 2022 Nov 3;11(21):3482. doi: 10.3390/cells11213482 (PMC9657739; doi:10.3390/cells11213482)
Supplement: Supplementary file 1 [file cells-11-03482-s001.zip › STables.pdf]

Supp. Table S1. Protein-Protein contacts between the principal residues[1,2] located on the *h*LIF loops and both *m*/*h*LIFr-LIF complex.

	*m*LIFr				*h*LIFr			
	Contacts			HBonds	Contacts			HBonds
*h*LIF	Good	Bad	Ugly		Good	Bad	Ugly	
P51	69	0	1	0	17	0	0	0
K153	3	0	0	0	10	0	0	0
F156	90	0	0	0	81	0	0	0
K159	37	6	2	1	41	2	0	0

Supp. Table S2. QPLD and IFD score values.

	QPLD	IFD		
RU486	*G-Score**	*G-Score**		*IFD-Score**
ID01	-5.427	-9.812		-10634.99
ID02	-4.772	A	-7.217	-10661.39
		B	-11.853	-10727.21

*Kcal/mol **Calculated with CPPTRAJ module after MD simulation.

Supp. Table S3. MM/GBSA values of the two most populated clusters.

MD Starting pose	#	Popn	MM/GBSA* ΔG^{tot}**
ID01	Cl0	43 %	-40.67 (±1.5)
	Cl1	30.4 %	-26.43 (±5.2)
ID02-B	Cl0	37.6 %	-18.51 (±1.5)
	Cl1	26.5 %	-28.54 (±3.2)

*Calculated with CPPTRAJ module. **Kcal/mol
