# Supplementary figures and images for: Repositioning Mifepristone as a Leukaemia Inhibitory Factor Receptor Antagonist for the Treatment of Pancreatic Adenocarcinoma
Source: Cells. 2022 Nov 3;11(21):3482. doi: 10.3390/cells11213482 (PMC9657739; doi:10.3390/cells11213482)

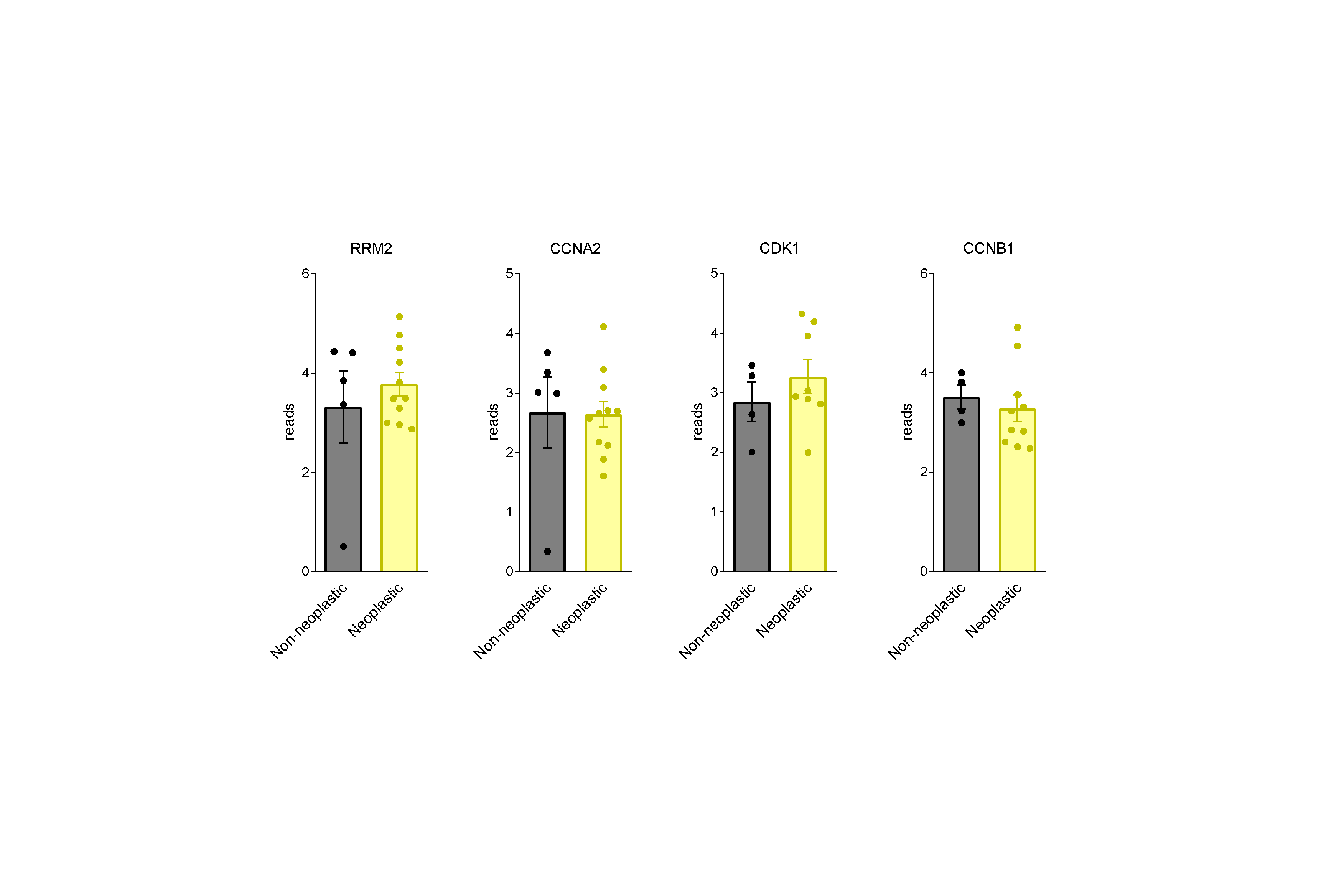

Supplement: Supplementary file 1 [file cells-11-03482-s001.zip › Figure S1.tif]

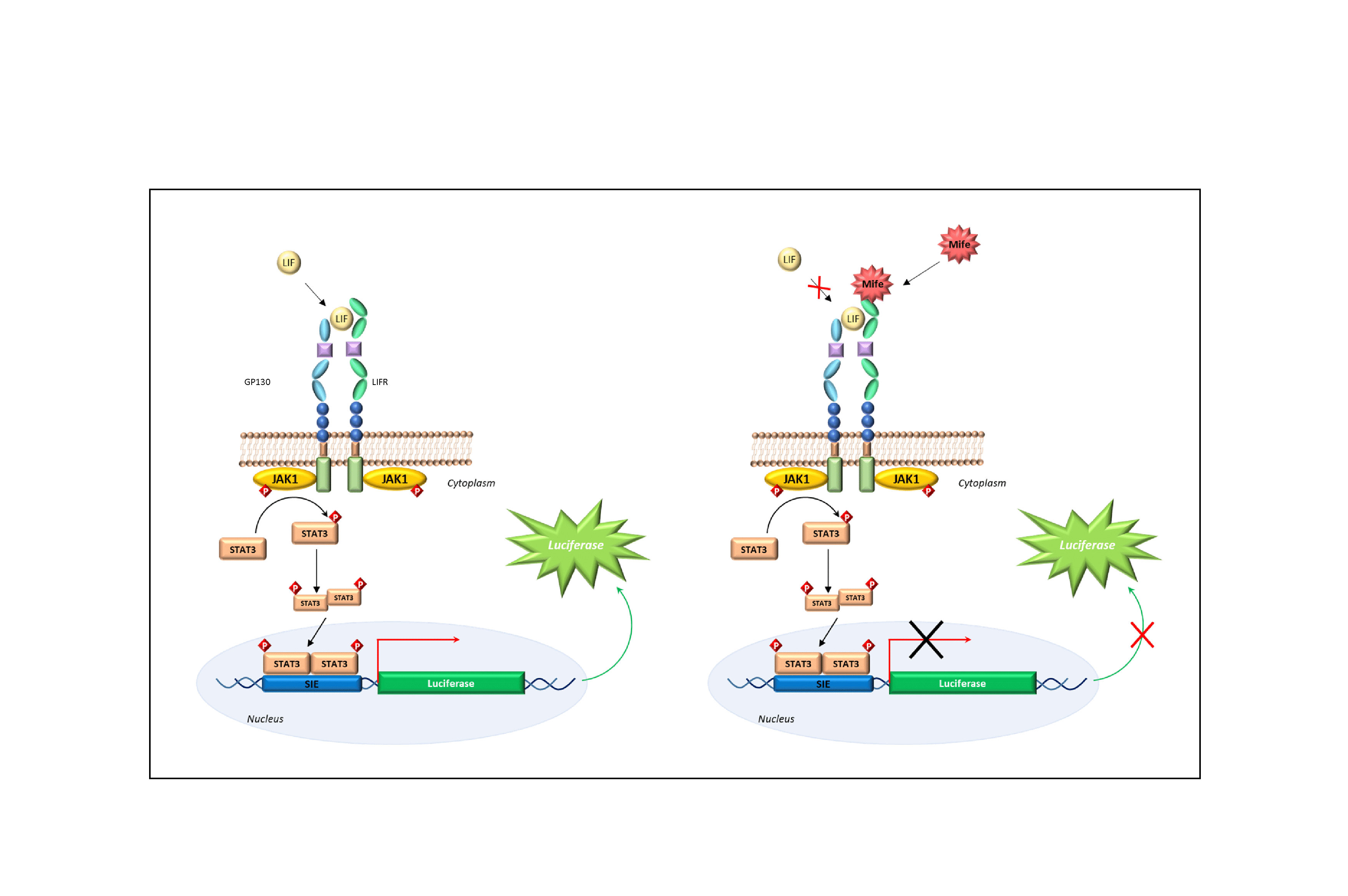

Supplement: Supplementary file 1 [file cells-11-03482-s001.zip › Figure S2.tif]

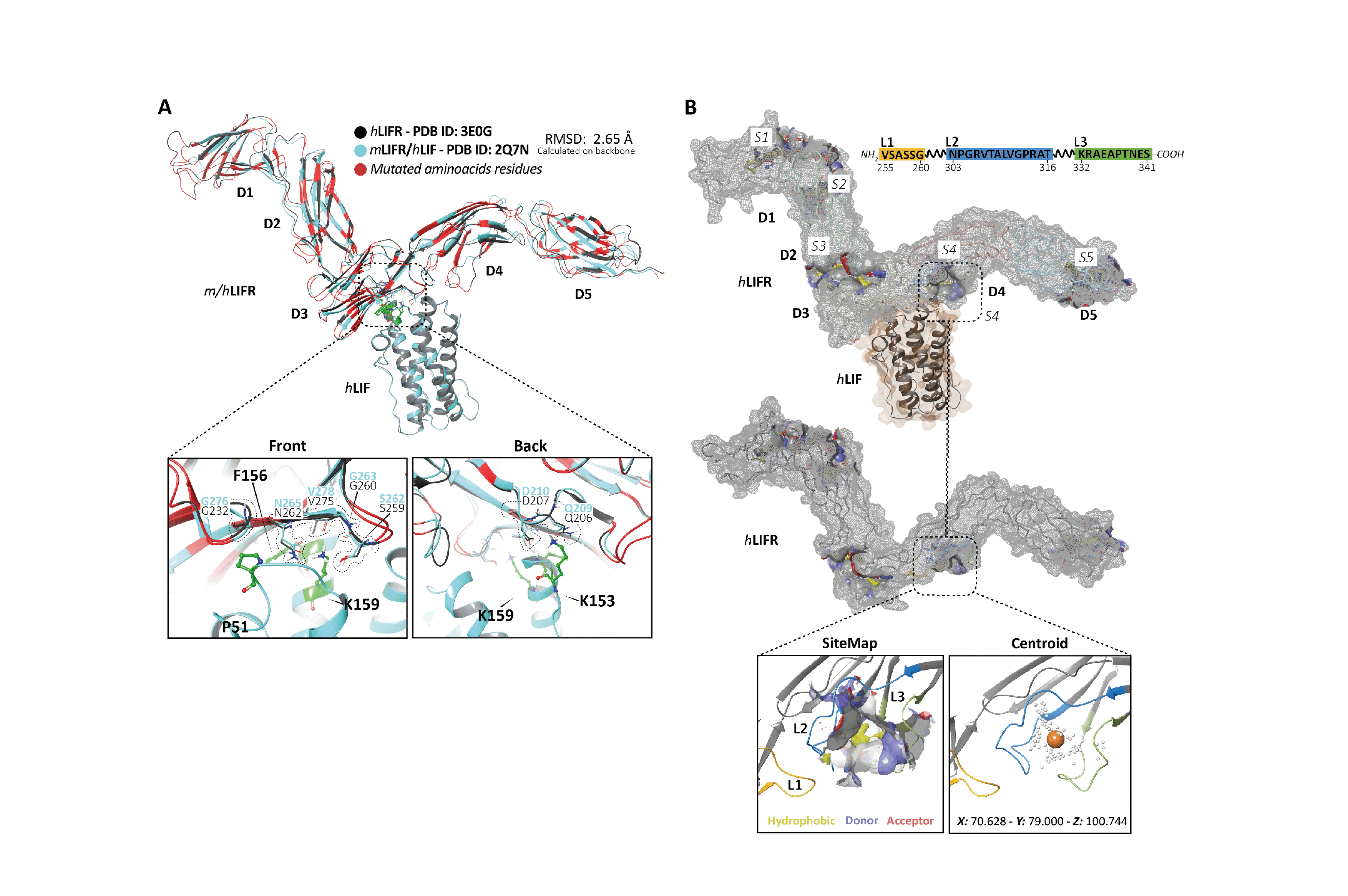

Supplement: Supplementary file 1 [file cells-11-03482-s001.zip › Figure S3.tif]

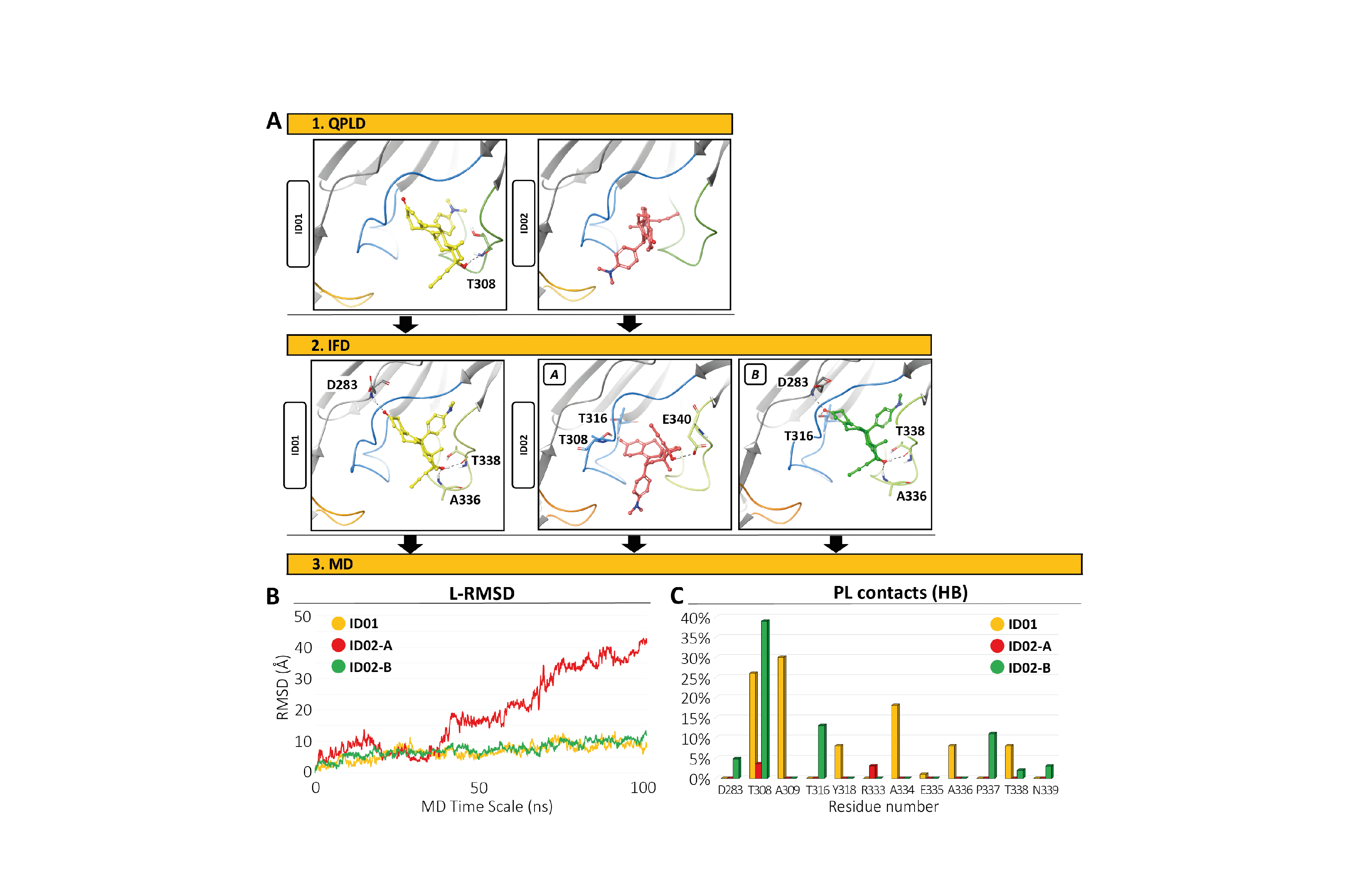

Supplement: Supplementary file 1 [file cells-11-03482-s001.zip › Figure S4.tif]

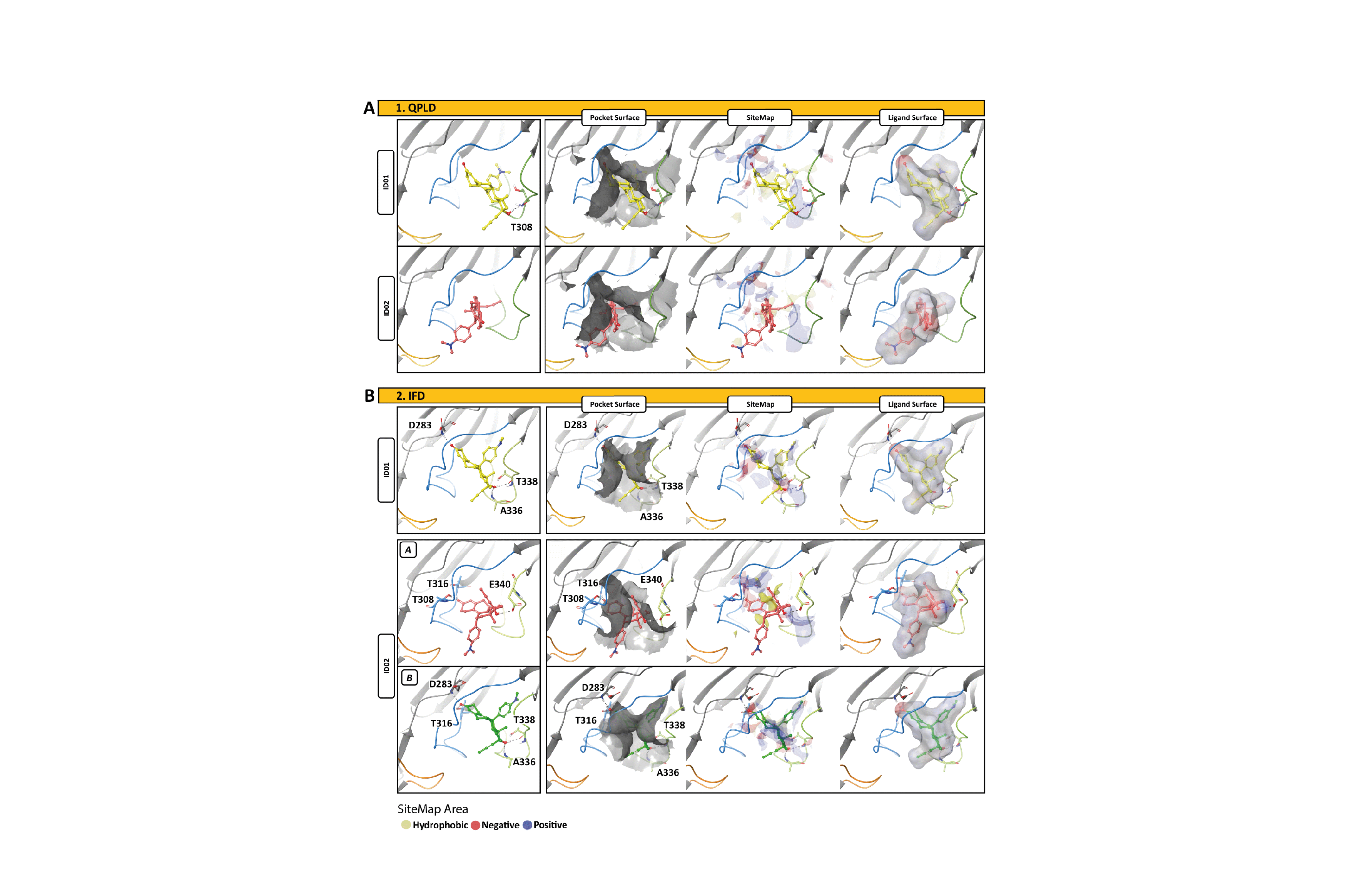

Supplement: Supplementary file 1 [file cells-11-03482-s001.zip › Figure S5.tif]

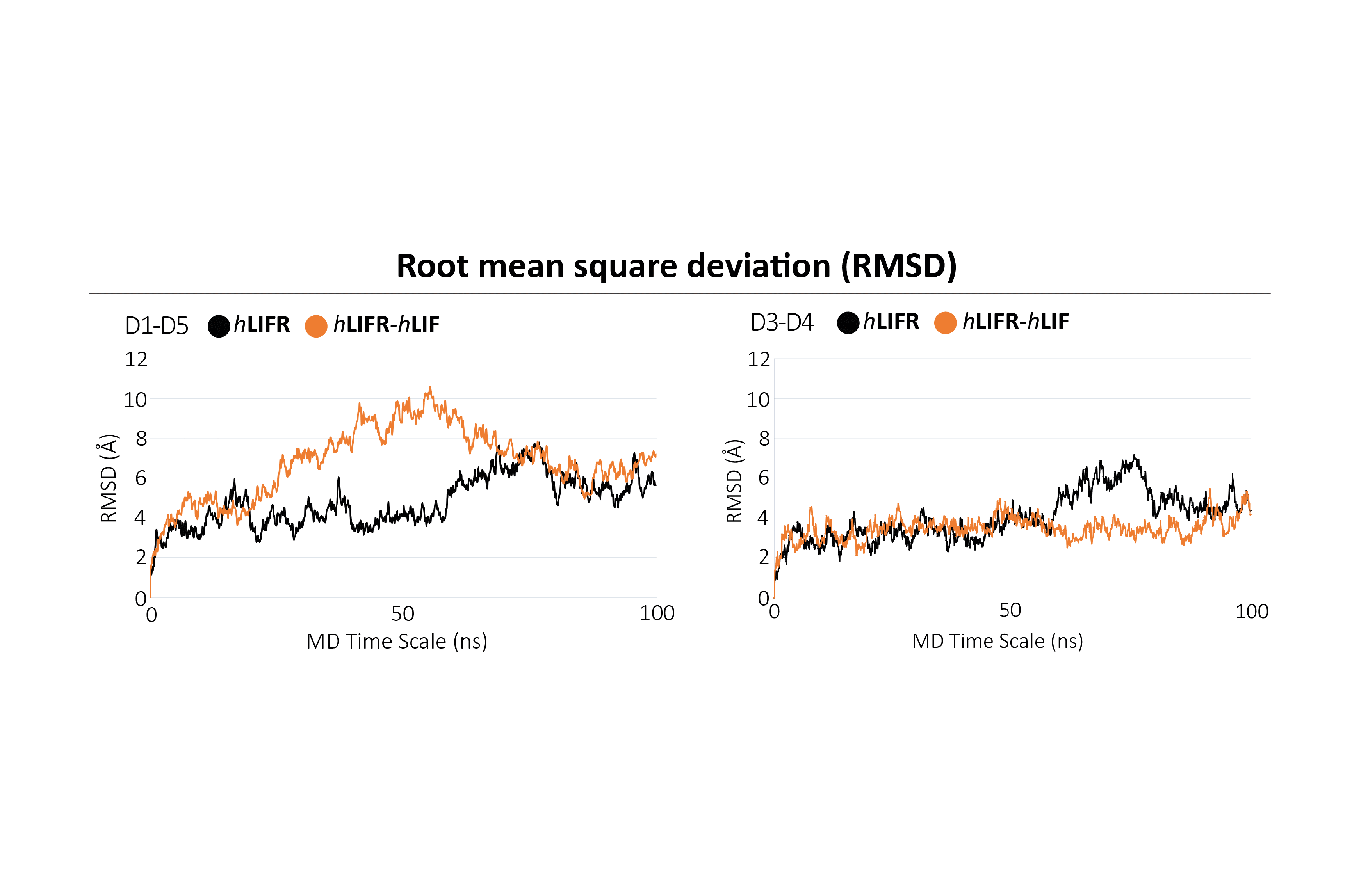

Supplement: Supplementary file 1 [file cells-11-03482-s001.zip › Figure S6.tif]

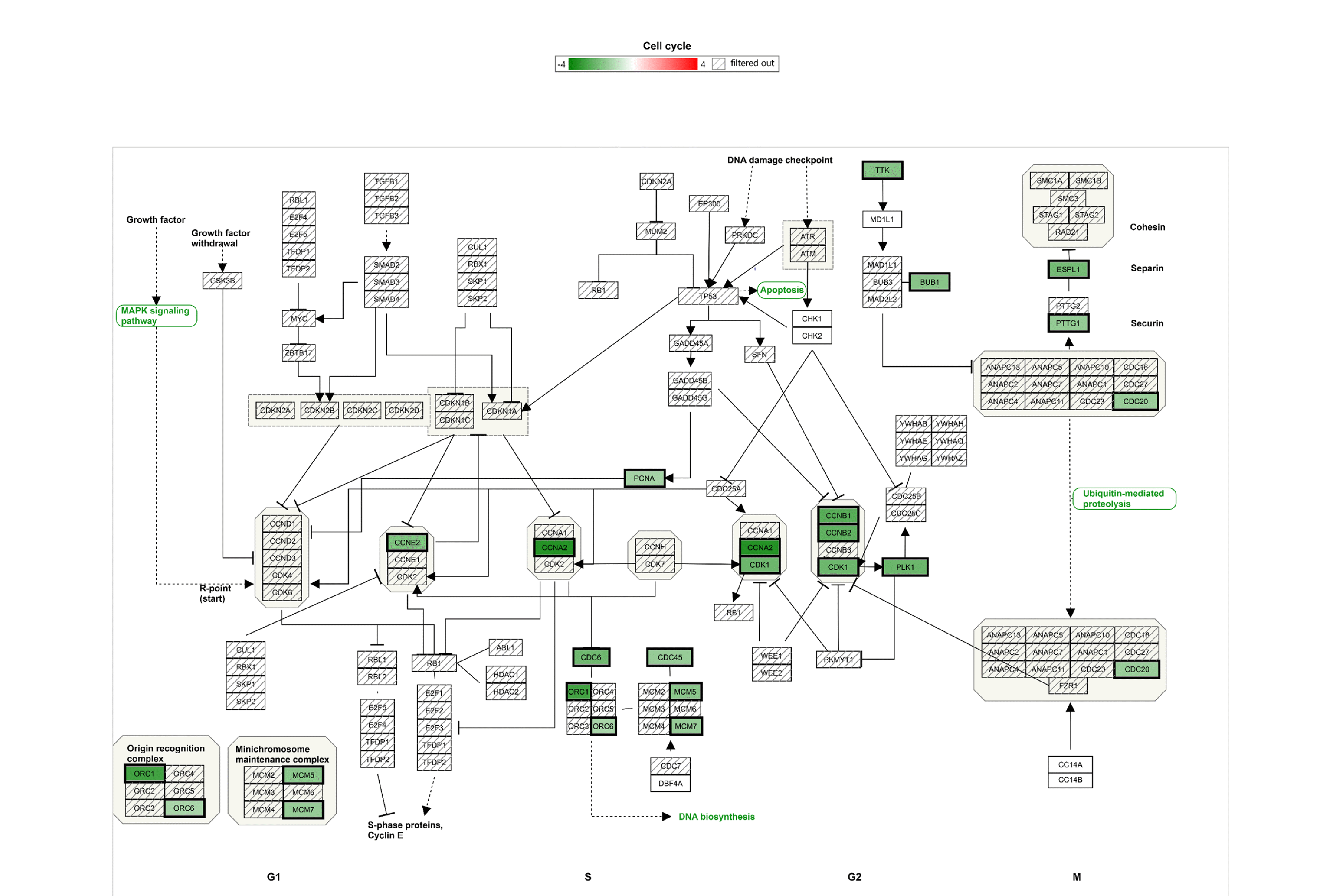

Supplement: Supplementary file 1 [file cells-11-03482-s001.zip › Figure S7.tif]
